# A Model for Bioaugmented Anaerobic Granulation

**DOI:** 10.3389/fmicb.2020.566826

**Published:** 2020-10-07

**Authors:** Anna Doloman, Amitesh Mahajan, Yehor Pererva, Nicholas S. Flann, Charles D. Miller

**Affiliations:** ^1^Department of Biological Engineering, Utah State University, Logan, UT, United States; ^2^Department of Computer Science, Utah State University, Logan, UT, United States

**Keywords:** anaerobic granulation, cDynoMiCs, bioaugmentation, agent-based model, biogas

## Abstract

Anaerobic granular sludge comprises of highly organized microorganisms with a sophisticated metabolic network. Such aggregates can withstand storage, temperature fluctuations and changes in the substrate supplied for anaerobic digestion. However, substrate change leads to long adaptation of granular consortia, creating lags in the reactor operations. To speed up adaptation and increase digestion efficiency, bioaugmentation with a robust consortium can be performed. The computational study described here aims to elucidate the mechanisms of bioaugmenting anaerobic granules, utilizing the current body of knowledge on metabolic and biochemical interactions between bacteria in such aggregates. Using a *cDynoMiCs* simulation environment, an agent-based model was developed to describe bioaugmentation for adaptation of cellobiose-degrading granular consortium to a lipid-rich feed. Lipolytic bacteria were successfully incorporated *in silico* to the stable granular consortia after 40 days of simulation. The ratio of cellobiose and the lipid-derivative, oleate, in the feed played key role to ensure augmentation. At 0.5 g/L of both cellobiose and oleate in the feed, a homogeneous stable augmented consortium was formed and converted the given amount of substrate to 10.9 mg/L of methane as a final product of anaerobic digestion. The demonstrated model can be used as a planning tool for anaerobic digestion facilities considering transition of the inoculum to a new type of feed.

## Introduction

Bioaugmentation is a common strategy in the field of wastewater treatment that is used to introduce a new metabolic capability to either aerobic or anaerobic microbial consortia (Stephenson and Stephenson, 1992; Dhouib et al., 2006; Herrero and Stuckey, 2015). A recent review (Nzila et al., 2016) pointed out applications of both yeast and bacterial bioaugmentations to treat various pollutants in wastewater: from azo-dyes to quinolines and polycyclic aromatic hydrocarbons. Success of the bioaugmentation is only possible if there is a substrate-specific niche available for the microbe to be incorporated into the already established consortia (Ellis et al., 2000; Da Silva and Alvarez, 2004; El Fantroussi and Agathos, 2005). Bioaugmentation shares the need for the substrate-specific niche with the concept of bioremediation, which often fails due to the lack of the unique metabolic niche (Liu and Suflita, 1993).

A number of studies demonstrated both successful and unsuccessful bioaugmentation when either substrate niche or pH favoring conditions were the limiting factors (Bouchez et al., 2000; Sträuber et al., 2015). For example, if during anaerobic digestion a compound is produced that is toxic or inhibitory to the intrinsic microbial community, incorporation of a novel microorganism that can remove the toxic/inhibitory compound would be beneficial (Schauer-Gimenez et al., 2010; Fotidis et al., 2013). Some research also suggests a need for tight biochemical interaction to take place between the bioaugmented bacterium and the intact community (Mohan et al., 2007; Schneider and Topalova, 2011). Such biochemical interactions, together with substrate niche availability, will lead to a stratification or compartmentalization of the bioaugmented bacterium in a densely packed microbial consortium. The best example of such densely packed microbial consortium is an anaerobic granule (Hulshoff Pol, 1989). Anaerobic granules are formed in upflow anaerobic sludge blanket (UASB) reactors, where due to the constant upflow velocity of the bottom-fed substrate and attraction toward some microbially-secreted polysaccharides (EPS), bacteria come together to form granules (Liu et al., 2003).

The study described here aims to shed light on the mechanisms of bioaugmenting anaerobic granules, utilizing the current state of knowledge on metabolic and biochemical interactions between bacteria in such aggregates. The end result of this study is a computational model that can visually demonstrate varying stratifications of different trophic microbial groups prior to and after bioaugmentation. This computer model can be useful for both researchers and engineers, who are operating or studying either laboratory or industrial-scale anaerobic digesters and wish to enhance rates of anaerobic decomposition and methane production via bioaugmentation.

In previous studies by our group, a model of *de novo* anaerobic granulation was successfully designed and a search engine was used to determine the optimum ratio of methanogenic and acidogenic bacteria, producing methane from the glucose-rich feed (Doloman et al., 2017). The new model reported here builds upon the basic principles of *de novo* anaerobic granulation reported earlier and introduces a more complex model of a granule with greater number of trophic groups. The described granule formation is based on the anaerobic decomposition of cellulose (in the form of a cellobiose) and is based on a larger microbial network of 5–6 different bacteria. Cellulose, being the main polysaccharide component of all plant and algal biomass, was chosen as a main model substrate due to its relevant biotechnological potential (Fu et al., 2010; Sawatdeenarunat et al., 2015; Kamali et al., 2016) and its relatively complex anaerobic digestion scheme (Weimer and Zeikus, 1977; Desvaux et al., 2001), allowing multiple microbial trophic groups to occupy the same layer in the granule.

To mathematically simulate the bioaugmentation process in UASB-like anaerobic digesters, new bacterial species are introduced to the matured cellobiose-fed granule, simultaneously with a new substrate that can only be decomposed by the newly introduced bacterium. A lipid derivative, oleate, was chosen as the alternative substrate that is degraded by the simulated bioaugmented granular consortium. Oleate is usually produced as an intermediate compound during anaerobic degradation of lipids by glycerol-fermenting acidogenic bacteria (Angelidaki et al., 1999). Oleate is introduced into the model together with an arbitrary oleate degrading bacterium, providing a metabolic contrast to the decomposition of the cellobiose. As a result, the model demonstrates bioaugmentation of the granule with new or additional metabolic capability. The selected cellulose-lipid combination of microbial substrates is a common anaerobically supplied feed in industries with mixed digestion profiles (Bohutskyi et al., 2015; Suksong et al., 2016). Initial microbial populations typically possess decomposition abilities toward only one part of the feed, but not to another (either cellulose or lipid). Thus, it usually takes months for the proper adaptation of the microbial consortia to decompose a mixed feed (Champion et al., 1999; Sreekrishnan et al., 2004; Hagman et al., 2008).

The study described here aims to shed light on the mechanisms of bioaugmenting anaerobic granules, utilizing the current state of knowledge on metabolic and biochemical interactions between bacteria in such aggregates. The end result of this study is a computational model that can visually demonstrate varying stratifications of different trophic microbial groups prior to and after bioaugmentation. This computer model can be useful for both researchers and engineers who are operating or studying either laboratory or industrial-scale anaerobic digesters and wish to enhance rates of anaerobic decomposition and methane production via bioaugmentation. The general aim of the study is to expand the knowledge on both successful bioaugmentation experiments and to inspire industrial-scale modifications in the anaerobic digestion processes.

## Materials and Methods

Models were developed in the *cDynoMiCs* agent-based simulator framework (Lardon et al., 2011). Initial predecessor of this framework, *iDynoMiCs*, was used to model biofilms. Both *c*-and *i*-versions of this framework assume cells as spherical particles, with given diameters. Each particle has it's own unique amount of associated biomass, cell growth and division characteristics, chemotactic species-specific instructions and an ability to form homogeneous/heterogeneous adhesion and associated tight junctions. A differential equation solver is implemented to compute the diffusion of supplied solutes (substrates and products), position of each particle with respect to the biochemical and biomechanical processes (such as secretion and uptake, adhesion and repulsion with the other particles in the system). All the solutes are assumed to be in a pseudo steady-state with respect to biomass growth. Once the solute (either gaseous or liquid) leaves the granular domain, it is no longer accounted for in the model. The model framework used in this current study is nearly identical to the one used in the previous *de novo* granulation model (Doloman et al., 2017) with some important modifications: addition of “sloughing function” and allowing biomass to decay when the substrate becomes unavailable. All the simulation details were specified in the XML protocol, providing instructions to be executed by the *cDynoMiCS* framework. *cDynoMiCS* writes plain-text XML files as output, and these may be processed using any number of software tools, such as Matlab and R. In addition to XML files, *cDynoMiCS* also writes files for POV-Ray to render 3D ray-traced images of the simulation. A domain size of 508 × 508 μm (2D) was used to run all the simulations. The modified *cDynoMiCs* can be found at the GitHub repository (https://github.com/adoloman/Modified-iDynoMICs-for-augmentation-model).

Seven solutes: cellobiose (*S*_*C*_), oleate (*S*_*O*_), lactate (*S*_*L*_), acetate (*S*_*A*_), ethanol (*S*_*E*_), hydrogen (*S*_*H*_), and methane (*S*_*M*_) exist within the reactor model. The distribution of these solutes is controlled by Equations 1, 2, 3, 4, 5, 6, and 7, respectively, where *S*_*i*_ denote concentration of the *i*-th solute, and *D*_*i*_ denotes correspondent solute diffusion value. *B*_*i*_ denotes biomass of the *i*-th bacterial cell type, μ_*i*_ describes correspondent bacterial type growth rate, while α_*bi*_ denotes biomass conversion rate. All the coefficients and their values for each of the equations here are presented in Supplementary Table 1.

The diffusion coefficients and reaction rates take different forms for each region depending upon the spatial distribution of six types of biomass: biomass of Clostridium1 (generic bacterium degrading cellobiose) (*B*_*c*1_), biomass of Clostridium2 (generic bacterium degrading lactate) (*B*_*c*2_), biomass of Oleate-degrader (*B*_*o*_), Desulfovibrio (generic bacterium degrading ethanol) (*B*_*d*_), and two types of methanogens (*B*_*m*2_), (*B*_*m*1_), degrading acetate and hydrogen, respectively. These relationships are described in the Equation 8. The effective diffusion coefficient is decreased within the granule compared with the liquid value in order to account for the increased mass transfer resistance (diffusion coefficient is multiplied by γ if the solute location coincides with presence of biomass at the same coordinates *x, y*). The diffusivity values used for the model (specified in Supplementary Table 1) are taken from literature related to biofilm diffusivity studies (Lens et al., 2003; Stewart, 2003).

(1) $$\begin{array}{rcl} \frac{\partial S_{C}}{\partial t} & = & B\left(x,y\right)\cdot D_{C}\cdot \frac{\nabla^{2}\;S_{C}}{\partial x\partial y}-\mu_{c1}\left(S_{C}\right)\cdot \frac{B_{c1}}{\alpha_{bc1}} \end{array}$$

(2) $$\begin{array}{rcl} \frac{\partial S_{O}}{\partial t} & = & B\left(x,y\right)\cdot D_{O}\cdot \frac{\nabla^{2}\;S_{O}}{\partial x\partial y}-\mu_{o}\left(S_{O},S_{A}\right)\cdot \frac{B_{o}}{\alpha_{bo}} \end{array}$$

(3) $$\begin{array}{rcl} \frac{\partial S_{L}}{\partial t} & = & B\left(x,y\right)\cdot D_{L}\cdot \frac{\nabla^{2}\;S_{L}}{\partial x\partial y}+\mu_{c1}\left(S_{C}\right)\cdot \frac{B_{c1}}{\alpha_{bc1}}\\ - & \mu_{c2}\left(S_{L}\right)\cdot \frac{B_{c2}}{\alpha_{bc2}} \end{array}$$

(4) $$\begin{array}{rcl} \frac{\partial S_{A}}{\partial t} & = & B\left(x,y\right)\cdot D_{A}\cdot \frac{\nabla^{2}\;S_{A}}{\partial x\partial y}+\mu_{d}\left(S_{E},S_{A}\right)\cdot \frac{B_{d}}{\alpha_{bd}}\\ + & \mu_{c2}\left(S_{L}\right)\cdot \frac{B_{c2}}{\alpha_{bc2}}-\mu_{m1}\left(S_{A}\right)\cdot \frac{B_{m1}}{\alpha_{bm1}} \end{array}$$

(5) $$\begin{array}{rcl} \frac{\partial S_{E}}{\partial t} & = & B\left(x,y\right)\cdot D_{E}\cdot \frac{\nabla^{2}\;S_{E}}{\partial x\partial y}+\mu_{c1}\left(S_{C}\right)\cdot \frac{B_{c1}}{\alpha_{bc1}}\\ - & \mu_{d}\left(S_{E},S_{A}\right)\cdot \frac{B_{d}}{\alpha_{bd}} \end{array}$$

(6) $$\begin{array}{rcl} \frac{\partial S_{H}}{\partial t} & = & B\left(x,y\right)\cdot D_{H}\cdot \frac{\nabla^{2}\;S_{H}}{\partial x\partial y}+\mu_{d}\left(S_{E},S_{A}\right)\cdot \frac{B_{d}}{\alpha_{bd}}\\ - & \mu_{m2}\left(S_{H}\right)\cdot \frac{B_{m2}}{\alpha_{bm2}} \end{array}$$

(7) $$\begin{array}{rcl} \frac{\partial S_{M}}{\partial t} & = & B\left(x,y\right)\cdot D_{M}\cdot \frac{\nabla^{2}\;S_{M}}{\partial x\partial y}+\mu_{m1}\left(S_{A}\right)\cdot \frac{B_{m1}}{\alpha_{bm1}}\\ + & \mu_{m2}\left(S_{H}\right)\cdot \frac{B_{m2}}{\alpha_{bm2}} \end{array}$$

where,

(8) $$B\left(x,y\right)=\{\begin{array}{l} 1.0\text{ if location x},\text{y contains no biomass}\\ \;\gamma \text{ if location x},\text{y contains biomass} \end{array}\;$$

Equations 9–14 describe changes in the biomass of all growing 6 bacterial cell types (Clostridium1, Clostridium2, Oleate-degraders, Desulfovibrio, and two types of methanogens) as a function of local cellobiose, acetate, lactate, ethanol, methane and hydrogen concentrations. A discrete switching mechanism is used to model cell death due to a lack of energy source. The switching mechanism is defined as the function *die*(*B*_*i*_) in the equations. For example, Clostridium1 cells are converted to dead cells when the amount of cellobiose is below a threshold value (death threshold in Supplementary Table 1) for a period of 96 h. Similarly, the Methanogen1 cells are converted to dead cells when the amount of acetate is below a threshold value (death threshold in Supplementary Table 1) for a period of 144 h. The rate of increase in dead cell mass is defined in Equation 15. The parameter values for controlling cell death are estimated due to the lack of studies quantifying the response of described cell types to nutritional stress.

(9) $$\begin{array}{rcl} \frac{\partial B_{c1}}{\partial t} & = & \mu_{c1}\left(S_{C}\right)B_{c1}-die\left(B_{c1}\right) \end{array}$$

(10) $$\begin{array}{rcl} \frac{\partial B_{c2}}{\partial t} & = & \mu_{c2}\left(S_{L}\right)B_{c2}-die\left(B_{c2}\right) \end{array}$$

(11) $$\begin{array}{rcl} \frac{\partial B_{o}}{\partial t} & = & \mu_{o}\left(S_{O},\;S_{A}\right)B_{o}-die\left(B_{o}\right) \end{array}$$

(12) $$\begin{array}{rcl} \frac{\partial B_{d}}{\partial t} & = & \mu_{d}\left(S_{E},\;S_{A}\right)B_{d}-die\left(B_{d}\right) \end{array}$$

(13) $$\begin{array}{rcl} \frac{\partial B_{m1}}{\partial t} & = & \mu_{m1}\left(S_{A}\right)B_{m1}-die\left(B_{m1}\right) \end{array}$$

(14) $$\begin{array}{rcl} \frac{\partial B_{m2}}{\partial t} & = & \mu_{m2}\left(S_{H}\right)B_{m2}-die\left(B_{m2}\right) \end{array}$$

(15) $$\begin{array}{rcl} \frac{\partial B_{dead}}{\partial t} & = & die\left(B_{c1}\right)+\;die\left(B_{c2}\right)+die\left(B_{o}\right)+die\left(B_{d}\right)\\ + & die\left(B_{m1}\right)+die\left(B_{m2}\right) \end{array}$$

The growth rates: of Clostridium1 is μ_*c*1_(*S*_*C*_), defined in Equation 16, the growth rate of Clostrodium2 is μ_*c*2_(*S*_*L*_), defined in Equation 17, the growth rate of Oleate-degraders is μ_*o*_(*S*_*O*_, *S*_*A*_), defined in Equation 18, the growth rate of Desulfovibrio is μ_*d*_(*S*_*E*_, *S*_*A*_), defined in Equation 19, the Methanogen1 is μ_*m*1_(*S*_*A*_) defined in Equation 20 and the growth rate of Methanogen2 is μ_*m*2_(*S*_*H*_), defined in Equation 21. In these equations, $\hat{\mu_{i}}$ is the maximum growth rate of the *i*-th cell type, *K*_*si*_ represent the *i*-th substrate saturation constant, while *K*_*ii*_ represent the *i*-th substrate inhibition constant.

From the equations, it can be observed that growth of Clostridium1, Clostridium2, and Methanogen2 follows Monod growth kinetic, while growth of Oleate-degraders has also product inhibition involved and both equations 19 and 20 for Desulfovibrios and Methanogen1 demonstrate Haldane growth kinetics with substrate and product inhibition. The Java code in *cDynoMiCs* was manipulated to add functionality of describing bacterial growth via Haldane kinetics.

(16) $$\begin{array}{rcl} \mu_{c1}\left(S_{C}\right) & = & \hat{\mu_{c1}}\cdot \frac{S_{C}}{K_{sC}+S_{C}} \end{array}$$

(17) $$\begin{array}{rcl} \mu_{c2}\left(S_{L}\right) & = & \hat{\mu_{c2}}\cdot \frac{S_{L}}{K_{sL}+S_{L}} \end{array}$$

(18) $$\begin{array}{rcl} \mu_{o}\left(S_{O},S_{A}\right) & = & \hat{\mu_{o}}\cdot \frac{S_{O}}{\left(K_{sO}+S_{O}\right)}\cdot \frac{K_{iAp}}{\left(K_{iAp}+S_{A}\right)} \end{array}$$

(19) $$\begin{array}{rcl} \mu_{d}\left(S_{E},S_{A}\right) & = & \hat{\mu_{d}}\cdot \frac{S_{E}}{\left(K_{sE}+S_{E}+S_{E}^{2}K_{ie}\right)}\cdot \frac{K_{iA}}{\left(K_{iA}+S_{A}\right)} \end{array}$$

(20) $$\begin{array}{rcl} \mu_{m1}\left(S_{A}\right) & = & \hat{\mu_{m1}}\cdot \frac{S_{A}}{\left(K_{sAc}+S_{A}+S_{A}^{2}K_{iAc}\right)} \end{array}$$

(21) $$\begin{array}{rcl} \mu_{m2}\left(S_{H}\right) & = & \hat{\mu_{m2}}\cdot \frac{S_{H}}{K_{sH}+S_{H}} \end{array}$$

The source code of *cDynoMiCs* was also modified to introduce a new “sloughing function,” which destroys all the granular biomass that grows above the set granule diameter. Sloughing is needed to simulate a UASB-like environment in the model. Granules in a UASB reactor are constantly under the sheer stress from the continuously flowing feed in the upflow mode. Thus, published works report a certain diameter threshold (2–3 mm), above which granules do not grow in the UASB-type reactor (Araya-Kroff et al., 2004; Nery et al., 2008). Current study uses a diameter of 500 μm (this number was mostly picked to decrease computational powers required to compute a bigger granule). The value of the maximum granular diameter is specified in the XML instructions. The sloughing function runs for every grid position in the simulation and determines whether a grid location should be destroyed or not, based on the XML-specified maximum diameter. A similar approach has been successfully implemented to model aerobic granular sludge (Xavier et al., 2007).

Instructions in the XML also include locations of the new species to be introduced to the already formed granule. When needed, new particles were supplied in the four corners of the square around core particle consortia. This study reports incorporation of additional bacterial species into the already formed granule. Instructions for additional supply of the species that will be incorporated are provided in the XML file, which can be found for each simulation part in the Github source code page provided below. Briefly, new species are introduced to the simulation environment by specifying their correspondent *x*, *y*, and *z* coordinates, for the location in close vicinity to the already formed granule. In all the simulations with incorporation of new species, those species were initially supplied in the four corners around the formed granule in the 508 × 508 μm (2D) domain. Within one cell doubling time, new species are captured within the outer surface of the growing granule. If there is enough growth substrate in the vicinity of those new cells, they multiply and spread within the depths of the granule. If substrate is scarce within the granule and cells cannot multiply further into the granule (following the gradient of the substrate), cells stay in the outer surface of the granule. Eventually, if growth substrate for the newly incorporated cells is scarce, cells from the outer layer of the granule will be sloughed off and no longer contribute to the simulation.

Finally, all of the simulations and results reported here were tested with varying numbers assigned to the “randomSeed” parameters in the XML of *cDynoMiCs*, to assess the effect of stochasticity on the model. Granular structure and cell distributions closely matched emerging behaviors among simulations with varying “randomSeed” starting numbers.

## Results

**Figure 1:**
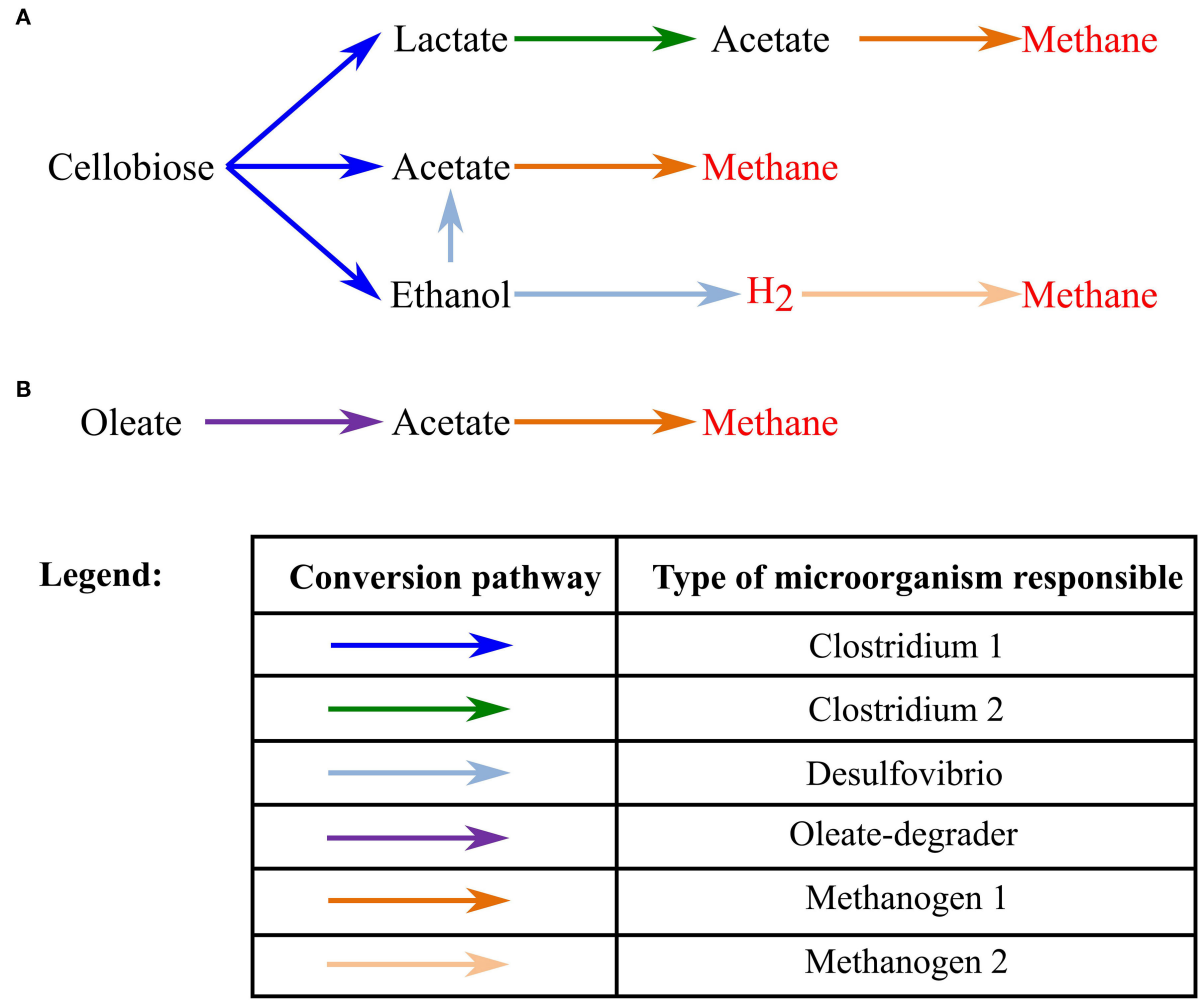
Schematic of the metabolic conversions in the studied anaerobic granules. **(A)** A pathway to convert cellobiose to the methane and hydrogen; **(B)** a pathway to convert oleate to methane.

In this study, we successfully developed and tested a model for bioaugmented anaerobic granules. Discussion of the results is divided into three main parts: (1) model of a granule grown on cellobiose; (2) model of a granule grown on cellobiose without ethanol-degrading bacteria, necessary to fully digest cellobiose, with augmentation at the later stages of granule development; and (3) model of a bioaugmented granule grown on oleate or a mix of oleate and cellobiose. An overall metabolic scheme for all simulation scenarios can be found in Figure 1.

### Formation of a Granule on Cellobiose

A granule with five types of bacteria (in scope of simulation called Clostridium1, Clostridium2, Desulfovibrio and two types of methanogens) was formed on constantly supplied cellobiose (1.5 g/L or 1 g/L), substrate for Clostridium1 cells. At 1.5 g/L concentration of cellobiose, all five types of bacterial cells grew on the products of cellobiose conversion into lactate, acetate and ethanol (Figure 1). On the contrary, 1 g/L of cellobiose was not sufficient to sustain growth of all four types of cells, leading to the decay of the lactate-fermenters, Clostridium2. There was 56% less of lactate produced from 1 g/L of cellobiose compared to 1.5 g/L of cellobiose, prior to the Clostridium2 decay at 144 hrs.

**Figure 2:**
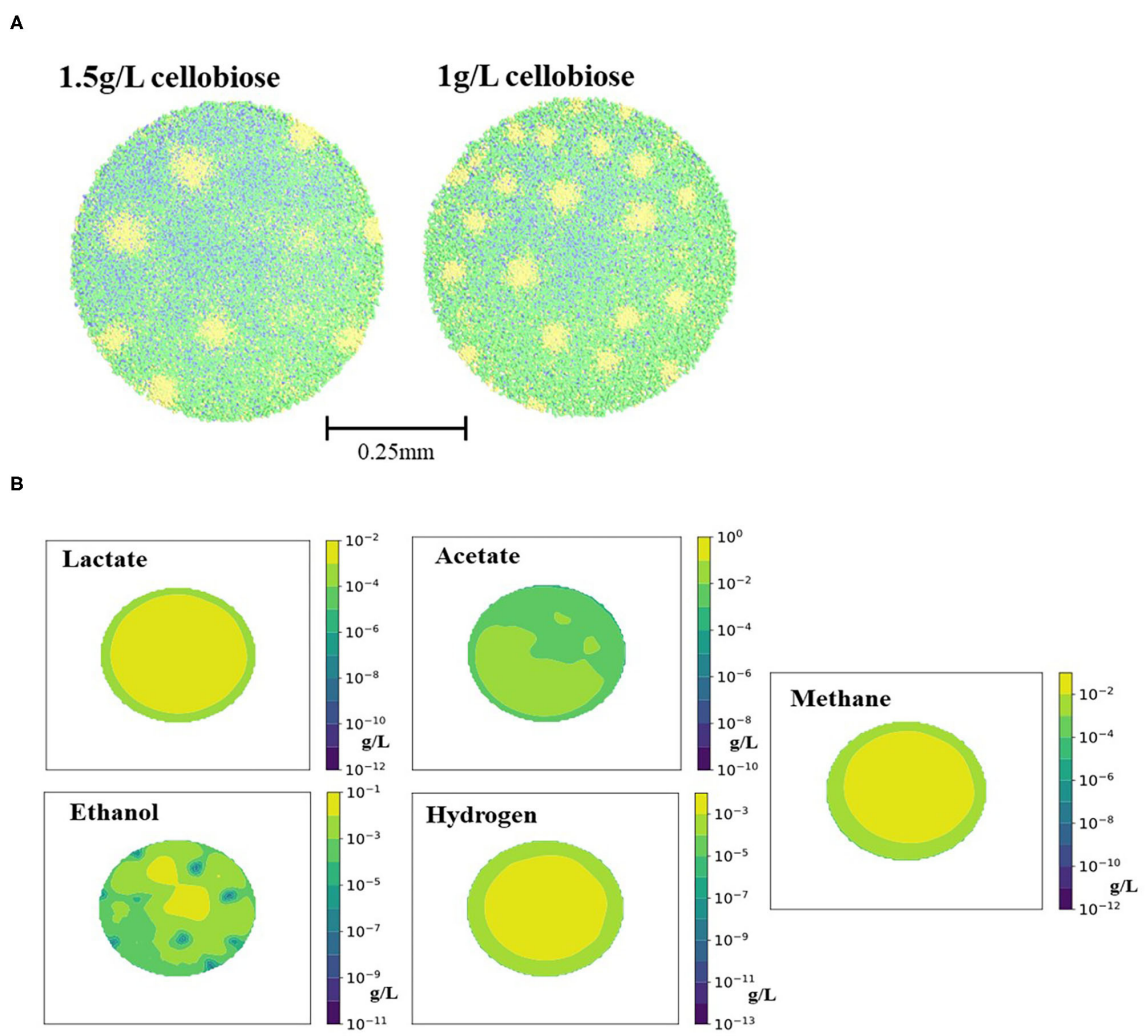
Images of **(A)** the spatial distribution of the microbial cell types in the granules grown on 1.5 and 1 g/L of cellobiose and **(B)** the correspondent spatial localizations of the 1.5 g/L cellobiose fermentation products (lactate, ethanol, acetate, hydrogen, and methane) on day 42 of simulation. Legend for **(A)**: green is Clostridium1, blue is Methanogen1 and Methanogen2, and yellow is Desulfovibrio. Legend for **(B)** corresponds to the colored scale of the concentration gradient next to each tile.

A 0.5 mm granule was formed after 700 h of computer simulation with both scenarios of cellobiose concentrations (corresponding to the 29 days in the lab-scale reactor). Steps of granule formation can be found on Supplementary Figure 1. After 29 days, the granule continued growth by radial expansion and peripheral cells were sloughed away. No particular stratification of different cell groups was observed (Figure 2A), except for the stratification of Desulfovibrio cells, converting ethanol to acetate and hydrogen. This cell type formed “pockets” inside the granule. The “pockets” map well to the ethanol distribution in the granule, as secreted by Clostridium1 cell types (Figure 2B). Absence of stratification for other cell types is different from the previous simulation of a glucose-fed granule (Doloman et al., 2017) and published laboratory studies (Rocheleau et al., 1999). Smooth diffusion gradient of the formed/consumed solutes can explain such cells distribution (Figure 2B). Such structure looks similar to the reported laboratory-studied granules fed with complex brewery, cellulose or protein-rich substrate (Batstone et al., 2004; Díaz et al., 2006; Baloch et al., 2008). Since all three initial cellobiose-derivatives (acetate, ethanol and lactate) were produced simultaneously, all three corresponding bacterial consumers (Clostridium2, Desulfovibrio, and Methanogen1) are present in the outer core of the granule and are present throughout the granule depth.

### Model of a Granule Augmented With Ethanol-Degrading Bacteria

**Figure 3:**
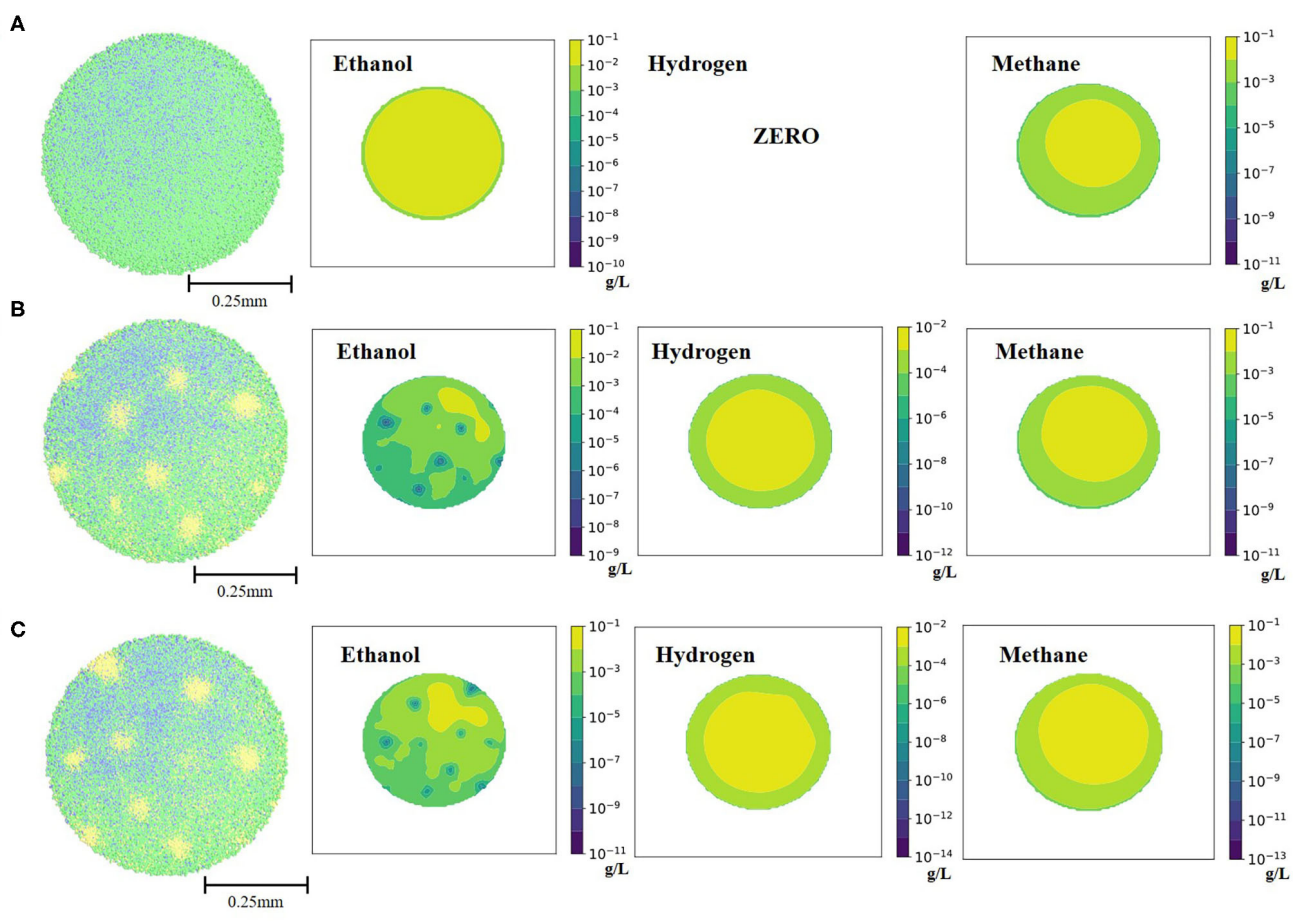
Spatial distribution of the bacterial cell types and three fermentation products at the end of the 42 days simulation for each ethanol-related scenario: **(A)** granule grown on 1.5 g/L of cellobiose, without ethanol-degraders; **(B)** granule with re-introduced ethanol-degraders after 16 days; **(C)** granule with re-introduced ethanol-degraders and hydrogenotrophic methanogens after 16 days. The three visible colors on the spatial distribution of the bacterial cell types are green (Clostridium1), blue (Methanogen1 and Methanogen2) and yellow (Desulfovibrio).

As previously stated, a key to bioaugmentation is availability of a substrate niche for a bacterium to be incorporated. To explore this *in silico*, ethanol-degrading Desulfovibrio was excluded from the simulation for 16 days and was re-introduced to the simulation environment later, on day 17. Accumulated ethanol (Figure 3) was readily available for the re-introduced Desulfovibrio and a successful augmentation was observed. It is important to note that in the current mathematical model set-up ethanol was not inhibitory to any of the cell types, except to the ethanol-degraders (see Materials and Methods for details). Thus, absence of a crucial mid-chain fermenter in the initial simulation for 16 days did not negatively affect all the cell types. The only cell type group that was negatively impacted by the absence of Desulfovibrio was Methanogen2 (bacteria that consume H_2_ from ethanol conversion). Consequently, the methane-producing potential of the granular consortia was decreased (Table 1). The next test scenario explored co-incorporation of both ethanol-degrading Desulfovibrio and hydrogenotrophic Methanogen2, to revive methane-generating potential of the granule. However, as can be seen from both Figure 3 and Table 1, re-introduction of Methanogen2 only slightly increased methane producing capacity of the granule, but for significant effects longer simulation will be needed.

**Table 1:** Final amounts of methane and hydrogen at the end of all simulation scenarios.

**Simulation scenarios (42 days)**	**Total methane produced, × 10^−10^, L**	**Total hydrogen produced, × 10^−10^, L**
1.5 g/L of cellobiose	9.29 (9.09 at 60 days)	5.8 (3.31 at 60 days)
1 g/L of cellobiose	3.65	2.77
Without Desulfovibrio^a^	5.16	0
With re-introduced Desulfovibrio after day 16^a^	6.81	5.47
With re-introduced Desulfovibrio and Methanogen2 after day 16^a^	7.02	5.3
With Oleate-degrader, 1.5 g/L oleate and 1.5 g/L of cellobiose	8.96	16.56
With Oleate-degrader, 1.5 g/L oleate, 1.5 g/L of cellobiose and 1 mm boundary granule growth	2.52	12.17
With Oleate-degrader, 1 g/L oleate and 1 g/L of cellobiose	5.37	3.31
With Oleate-degrader, 0.5 g/L oleate and 0.5 g/L of cellobiose	3.04 (22.43 at 60 days)	0.66 (1.44 at 60 days)
With Oleate-degrader and 1.5 g/L oleate	0.21 (23.13 at 60 days)	0

a *cellobiose concentration in the feed was 1.5 g/L*.

### Model of a Bioaugmented Granule Grown on Oleate or a Mix of Oleate and Cellobiose

To investigate the possibility of incorporating a new bacterium type into the cellobiose-fed granule, a lipid-degrading bacterium was chosen. Scenarios with and without substrate pressure were investigated.

#### Augmentation When Both Oleate and Cellobiose (1.5, 1, and 0.5 g/L Scenarios) Are Available in the Environment

**Figure 4:**
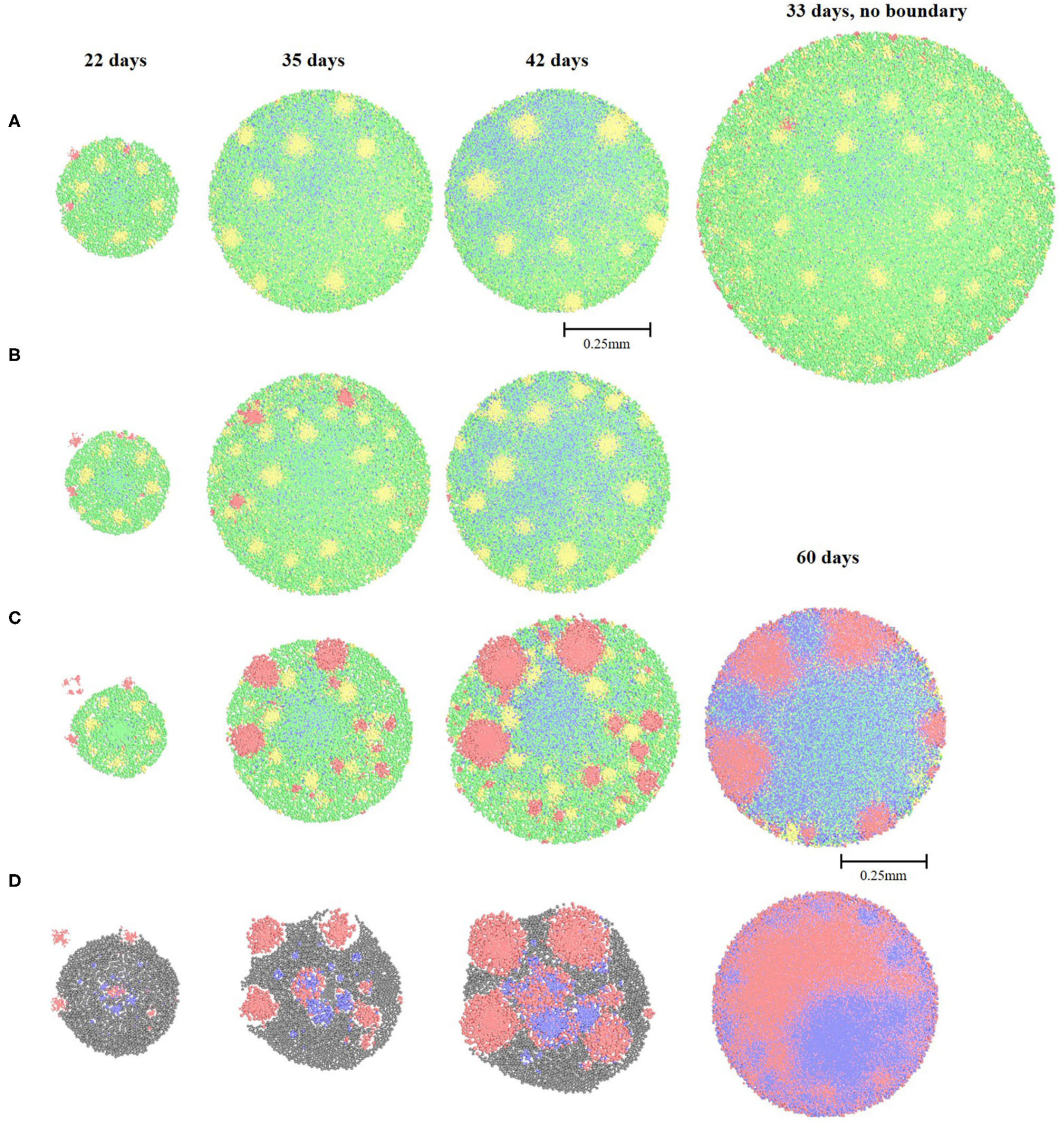
Spatial distribution of the bacterial cell types and fermentation products throughout the incorporation experiment with Oleate-degraders. **(A)** granule grown on 1.5 g/L of cellobiose and oleate; **(B)** granule grown on 1 g/L of cellobiose and oleate; **(C)** granule grown on 0.5 g/L of cellobiose and oleate; **(D)** granule grown on 1.5 g/L of oleate, cellobiose supply is halted at the time of incorporation on day 17. The color legend: green (Clostridium1), blue (Methanogen1 and Methanogen2), yellow (Desulfovibrio) and red (Oleate-degraders).

Augmentation of Oleate-degraders with both oleate and cellobiose substrates was differently influenced by the varying concentrations of oleate and cellobiose (Figure 4). Augmentation with oleate-degraders was simulated on 17-days-old cellobiose-degrading granule. With 1.5 g/L of both substrates Oleate-degraders were incorporated into the granule only during the first 12 days of simulation, until the growth limit of 0.5 mm was reached. After that all the newly-incorporated Oleate-degraders were steadily pushed to the outer layers of the granule and sloughed off the granule surface (Figure 4A). Similar results from bioaugmenting anaerobic consortia with lipolytic bacteria were reported by Cirne and colleagues (Cirne et al., 2006). In the described study bioaugmented bacterium did not stay for the whole duration of the anaerobic digestion, and was detected by the T-RFLP only at the beginning of the experiment. This might have been due to the similar washout as reported in this simulated study.

If the “sloughing function” (see Materials and Methods) is turned off in our model and the granule diameter is allowed to increase by 40%, Oleate-degraders are incorporated into the outer layers and into some scattered locations inside the granule (Figure 4A, 33 days). This observation can support the need for a reduced flow rate in a UASB reactor during the bioaugmentation period, allowing granules to grow bigger with less turbulent sloughing of the outer granular layers and slower washout of the non-incorporated bacteria. In addition, allowing peripheral granular growth may be critical if the bioaugmented species are of importance for the primary hydrolysis of a supplied substrate.

Decreasing concentration of both substrates to 1 g/L slowed down the sloughing of the Oleate-degraders, but after 42 days of simulation only a few cells of that type can be observed in the very outer layers (Figure 4B). Further decrease in the substrate concentration down to 0.5 g/L finally lead to the complete incorporation of the Oleate-degraders into the granular consortia and produced a very homogeneous structure (Figure 4C). Methane production in such augmented granule was significantly increased to 10.86 mg/L on day 60.

#### Augmentation With Only 1.5 g/L Oleate Present in the Environment

When lipid derivative, oleate, was used as a sole feed for the established granule on cellobiose, Oleate-degraders were successfully incorporated into the granule, but all other cell types decayed, due to the lack of cellobiose fermentation products (Figure 4D). The only other cell type that survived was acetoclastic Methanogen1, feeding off acetate produced from oleate by Oleate-degraders. Methanogen1 cell types exhibited “pocketing” behavior, growing at the places where acetate was previously supplied to them by Clostridium1 and ethanol-degrading Desulfovibrio. Similar behavior for acetoclastic methanogenic bacteria in anaerobic granules was previously reported (Schmidt and Ahring, 1999; Liu et al., 2002). Methanogens benefitted from the change in the microbial composition of the augmented granule: despite the initial drop in methane production after 42 days, there was a drastic increase after 60 days: 11.2 mg/L of methane (Table 1). Such amount of methane is far higher than that of a granule grown on cellobiose alone for 60 days (4.4 mg/L), where methanogens are the terminal acceptors of acetate and hydrogen after a multiple step conversion of cellobiose.

**Figure 5:**
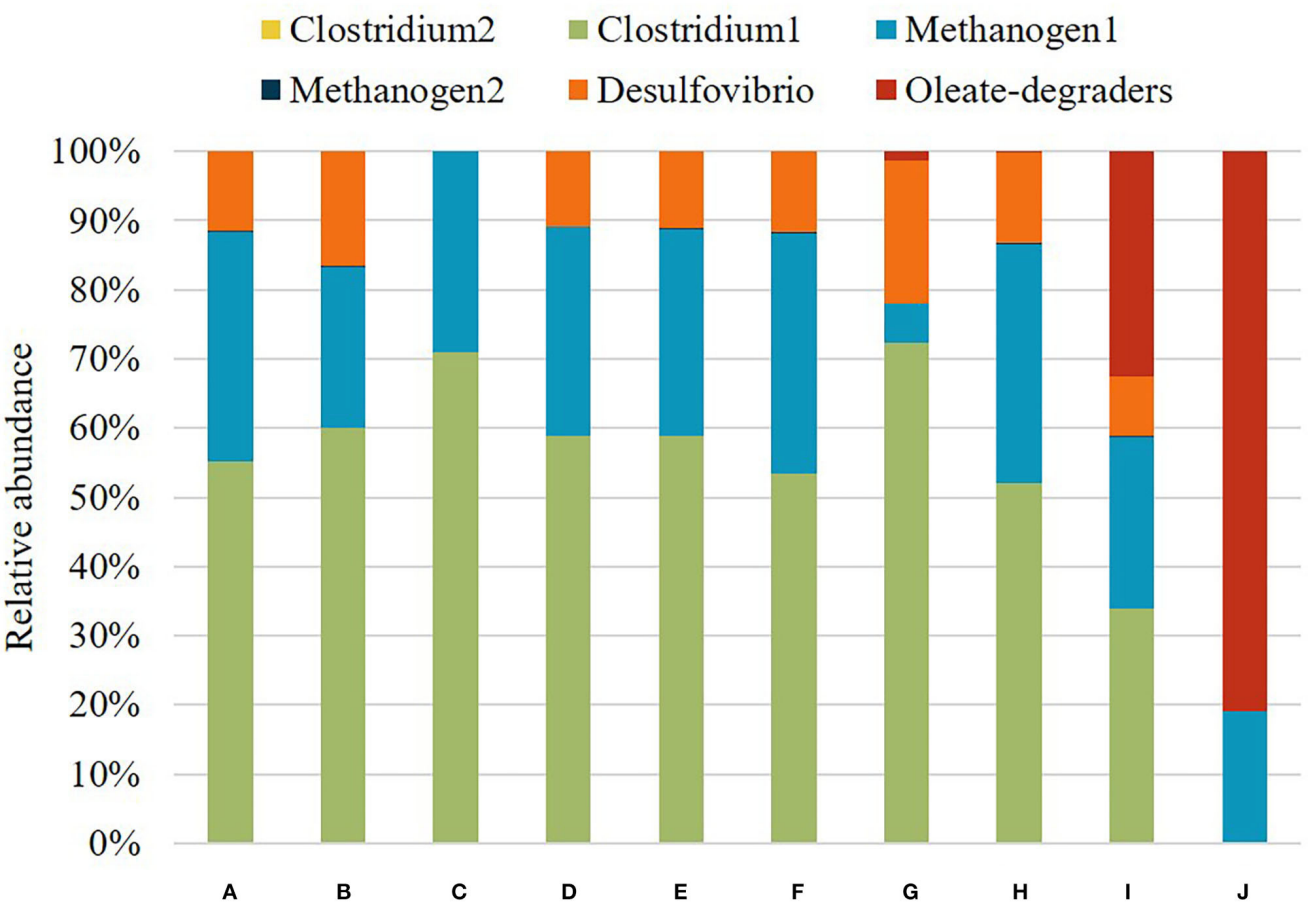
Cell type composition of each granule in different simulation scenarios. **(A)** 1.5 g/L cellobiose, **(B)** 1 g/L cellobiose, **(C)** 1.5 g/L cellobiose without ethanol-degrading Desulfovibrio, **(D)** 1.5 g/L cellobiose with re-introduced Desulfovibrio on day 16, **(E)** 1.5 g/L cellobiose with re-introduced Desulfovibrio and Methanogen2 on day 16, **(F)** 1.5 g/L oleate and 1.5 g/L of cellobiose with Oleate-degraders, **(G)** 1.5 g/L oleate and 1.5 g/L of cellobiose with Oleate-degraders and 1 mm boundary granule growth, **(H)** 1 g/L oleate and 1 g/L of cellobiose with Oleate-degraders, **(I)** 0.5 g/L oleate and 0.5 g/L of cellobiose with Oleate-degraders, **(J)** 1.5 g/L oleate with Oleate-degraders.

Another peculiarity is the black biomass in the augmented granule (Figure 4D), which is a dead cell mass formed due to the substrate shift. Such a high amount of dead biomass can lead to the breakdown of granules in real UASB reactors and formation of smaller “daughter” granules, only with two cell types: Oleate-degraders and Methanogen1 (Grotenhuis et al., 1991). However, this division should only occur under a sheer stress of the upflow velocity in the UASB reactors, when the flow is high enough to physically break the granule with dead particles in it (Mu and Yu, 2006). Otherwise, newly augmented granule will continue to grow with cavities, just like predicted in our model (Figure 4D) and as described in laboratory studies (Schmidt and Ahring, 1999; Liu et al., 2002; Muda et al., 2013). Summary of all cell types distribution at the end of all simulation scenarios can be found in Figure 5.

## Discussion

The model for a bioaugmented granule presented here was successfully developed in the agent-based simulator framework, *cDynoMiCs*. Demonstrated results support substrate-niche necessity for successful bioaugmentation. In addition, results demonstrate importance of considering the type of feed that is used during bioaugmentation. A unique combination of new and old substrates is needed, to support growth of all bacterial species, including all of those already existing in the granular consortia and the ones to be incorporated into the granule. Low concentrations of substrates that support bacteria to be augmented can make those new species highly prone to sloughing off the surfaces of the granules, with inability to be incorporated into the inner layers of the granule. More research is needed to find the exact ratio of augmenting substrate to the previously used one and algorithms can help to screen the area of parameters *in silico* (Doloman et al., 2017). Also, more investigations are needed to be done on the importance of granular sloughing diameter, strength of the feed in the simulated UASB reactors, and correspondent washout speeds.

The computational study described here also supports experimental observations by (Rocheleau et al., 1999; Batstone et al., 2004; Díaz et al., 2006; Baloch et al., 2008) in visualizing stratification/lack of thereof in a complex substrate-fed granule. The main conclusion is that stratification in granules is more likely to occur if solutes (substrates and products governing biochemical conversions) have varying diffusion coefficients, thus not being homogeneously available to all the correspondent microbial consumers. In addition to stratification, this *in silico* study provides an explanation for the reasons some anaerobic granules are seen with cavities (dead bacterial biomass) in them (Schmidt and Ahring, 1999; Liu et al., 2002; Muda et al., 2013): a combination of low flow rate inside UASB reactor and low diffusivity of the growth supporting substrate into the core of the granule will lead to the decay of bacterial biomass. Switching to the higher flow rates in the bioreactors can lead to division of cavitated granules into smaller granules with continuation of their growth and expansion.

The described model can be further extended and applied to test various combinations of microorganisms and changing substrate feeds. Based on the reported results above, the model produces reliable, predictable and literature-validated observations. The model still needs improvements on both the framework and biological side. Potential additions to the simulator code will include an algorithm to simulate division of a mature complex granule into two daughter granules, exploring a scenario of a complete substrate switch, and sudden biomass decay. In addition, the model needs improvements from the biological and reactor operations stand points. For example, adding complexity into the microbial interactions via flow of electron-donors and electron-acceptors between separate cells (such as sulfates and oxygen). Simulation of how anaerobic system can adapt to the trace amounts of oxygen present during the start-up of the reactors and resulting microbial fluctuations can bring some useful insights into operation of the anaerobic reactors under varying feed conditions.

Potential future application of the *cDynoMiCs* framework demonstrated here will be in adding a functionality in the code to model granulation with addition of the granulating agents, such as calcium and magnesium ions, or even activated carbon. Of particular interest is development of a model that can describe mechanisms of saline wastewater anaerobic digestion. As reported in the recent studies (Gagliano et al., 2017; Sudmalis et al., 2018), sodium ions can replace calcium ions inside the granule but not necessarily lead to the disruption of the aggregates. Since the mechanisms of the described process are not exactly clear, a computer model might be useful in that area.

Overall, modeling of anaerobic granulation during bioaugmentation process proved useful in visually demonstrating the importance of the substrate niche and impact of washout on the outcome of digestion enhancement. The current model can be used as a great planning tool for researchers assessing the potential of bioaugmentation strategies for the known consortia in their anaerobic reactors, thus eliminating the risk of crushing the reactor due to the improper planning.

## Data Availability Statement

The datasets presented in this study can be found in online repositories. The names of the repository/repositories and accession number(s) can be found at: https://github.com/adoloman/Granular-augmentation-model.

## Author Contributions

AD conceived the study and together with AM developed the plan for the simulation. AM updated the code for the framework and ran simulations. YP developed a code for image processing and analysis. AD wrote the manuscript. NF and CM supervised the work and reviewed the manuscript. All authors contributed to the article and approved the submitted version.

## Conflict of Interest

The authors declare that the research was conducted in the absence of any commercial or financial relationships that could be construed as a potential conflict of interest.
