# Supplementary material for: A Model for Bioaugmented Anaerobic Granulation
Source: Front Microbiol. 2020 Oct 7;11:566826. doi: 10.3389/fmicb.2020.566826 (PMC7575707; doi:10.3389/fmicb.2020.566826)
Supplement: Supplementary file 1 [file Data_Sheet_1.PDF]

Supplementary Material to "A Model for Bioaugmented Anaerobic Granulation"

Anna Doloman, Amitesh Mahajan, Yehor Pererva, Nicholas S. Flann, Charles D. Miller

1 Supplementary Figures and Tables

1.1 Supplementary Figures

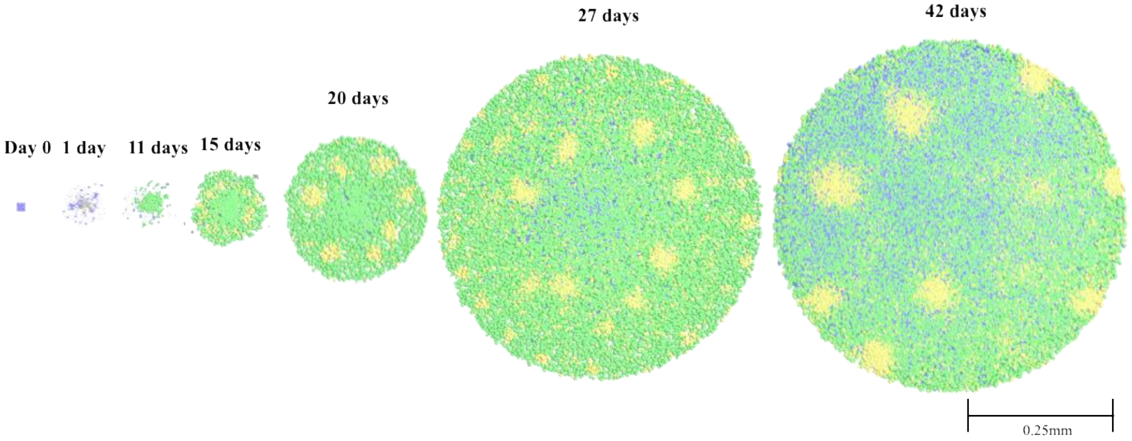

Supplementary Figure 1. Stages of granule formation on 1.5 g/L of cellobiose. Color legend for cell types: green (cellobiose-degrading clostridium1), red (lactate-degrading Clostridium2), yellow (ethanol-degrading Desulfovibrio), blue (acetoclastic and hydrogenotrophic methanogens).

1.2 Supplementary Tables

Supplementary Table 1. Parameters used to run the simulation models in *iDynoMiCs.*

Model parameter	Symbol	Value	Unit	References
Diffusion of cellobiose in liquid	D_C	5.72×10^{-5}	m^2/day	(Ihnat and Goring, 1967)
Diffusion of oleate in liquid	D_O	3.1×10^{-3}	m^2/day	(Stewart et al., 1991)
Diffusion of lactate in liquid	D_L	6.22×10^{-5}	m^2/day	(Hazel and Sidell, 1987)
Diffusion of acetate in liquid	D_A	1.34×10^{-4}	m^2/day	(Hobbie and Roth, 2007)
Diffusion of ethanol in liquid	D_E	9.3×10^{-5}	m^2/day	(Cussler, 2009)
Diffusion of hydrogen in liquid	D_H	4.98×10^{-4}	m^2/day	(Cussler, 2009)
Diffusion of methane in liquid	D_M	1.65×10^{-4}	m^2/day	(Haynes, 2012)
Biofilm diffusivity	γ	30	%	(Lens et al., 2003)
Clostridium 1				
Cell mass	B_{c1}	500	fg	(Kubitschek, 1990)
Division radius		2	µm	estimated

Maximum growth rate	$\widehat{\mu_{c1}}$	0.15	h^{-1}	(Guedon et al., 1999; Desvaux et al., 2001)
Substrate saturation constant	K_{sC}	2.5	g/L	(Guedon et al., 1999; Desvaux et al., 2001)
Biomass conversion rate	α_{bc1}	0.203	$\frac{g_{biomass}}{g_{cellobiose}}$	(Guedon et al., 1999; Desvaux et al., 2001)
Substrate conversion rate	α_{ac1}	0.45	$\frac{g_{acetate}}{g_{cellobiose}}$	(Guedon et al., 1999; Desvaux et al., 2001)
Substrate conversion rate	α_{lc1}	0.0096	$\frac{g_{lactate}}{g_{cellobiose}}$	(Guedon et al., 1999; Desvaux et al., 2001)
Substrate conversion rate	α_{ec1}	0.28	$\frac{g_{ethanol}}{g_{cellobiose}}$	(Guedon et al., 1999; Desvaux et al., 2001)
Death delay		96	h	estimated
Death threshold		0.02	g/L	estimated
OleateDegrader				
Cell mass	B_o	500	fg	(Kubitschek, 1990)
Division radius		2	µm	estimated
Maximum growth rate	$\widehat{\mu_o}$	0.1	h^{-1}	(Angelidaki et al., 1999)
Substrate saturation constant	K_{sO}	0.02	g/L	(Angelidaki et al., 1999)
Product inhibition constant	K_{iAp}	5	g/L	(Angelidaki et al., 1999)
Biomass conversion rate	α_{bo}	0.1	$\frac{g_{biomass}}{g_{oleate}}$	(Angelidaki et al., 1999)
Substrate conversion rate	α_{ao}	1.85	$\frac{g_{acetate}}{g_{oleate}}$	(Angelidaki et al., 1999)
Death delay		96	h	estimated
Death threshold		0.00002	g/L	estimated
Clostridium 2				
Cell mass	B_{c2}	500	fg	(Kubitschek, 1990)
Division radius		2	µm	estimated
Maximum growth rate	$\widehat{\mu_{c2}}$	0.144	h^{-1}	(Yang and Tang, 1991)
Substrate saturation constant	K_{sL}	0.03	g/L	(Yang and Tang, 1991)
Biomass conversion rate	α_{bc2}	0.06	$\frac{g_{biomass}}{g_{lactate}}$	(Yang and Tang, 1991)
Substrate conversion rate	α_{al}	0.98	$\frac{g_{acetate}}{g_{lactate}}$	(Yang and Tang, 1991)
Death delay		144	h	estimated
Death threshold		0.00001	g/L	estimated
Desulfovibrio				
Cell mass	B_d	500	fg	(Kubitschek, 1990)
Mass of EPS capsule		10	fg	estimated

Division radius		2	μm	(Tatton et al., 1989; Seitz et al., 1990)
Maximum growth rate	$\widehat{\mu_d}$	0.125	h^{-1}	(Tatton et al., 1989; Kaksonen et al., 2003)
Substrate saturation constant	K_{sE}	0.00045	g/L	(Nagpal et al., 2000)
Product inhibition constant	K_{iA}	7.2	g/L	(Seitz et al., 1990; Kaksonen et al., 2003)
Substrate inhibition constant	K_{ie}	80.5	g/L	(Tatton et al., 1989; Seitz et al., 1990)
Biomass conversion rate	α_{bd}	0.22	$\frac{g_{biomass}}{g_{ethanol}}$	(Tatton et al., 1989; Seitz et al., 1990; Nagpal et al., 2000)
Substrate conversion rate	α_{ac}	1.3	$\frac{g_{acetate}}{g_{ethanol}}$	(Tatton et al., 1989; Seitz et al., 1990; Nagpal et al., 2000)
Substrate conversion rate	α_{hc}	0.17	$\frac{g_{hydrogen}}{g_{ethanol}}$	(Tatton et al., 1989; Seitz et al., 1990; Nagpal et al., 2000)
Death delay		120	h	estimated
Death threshold		0.000001	g/L	estimated
Methanogen 1				
Cell mass	B_{m1}	1000	fg	(Sowers et al., 1984)
Mass of EPS capsule		10	fg	(Moletta et al., 1986)
Division radius		2	μm	(Sowers et al., 1984)
Maximum growth rate	$\widehat{\mu_{m1}}$	0.1	h^{-1}	(Koesnandar et al., 1990)
Substrate saturation constant	K_{sAc}	0.005	g/L	(Moletta et al., 1986)
Substrate inhibition constant	K_{iAc}	0.24	g/L	(Ibba and Fynn, 1991; Gavala et al., 2003)
Biomass conversion rate	α_{ba}	0.15	$\frac{g_{biomass}}{g_{acetate}}$	(Koesnandar et al., 1990; Kalyuzhnyi and Davlyatshina, 1997)
Substrate conversion rate	α_{ma}	0.26	$\frac{g_{methane}}{g_{acetate}}$	(Koesnandar et al., 1990)
Death delay		144	h	estimated
Death threshold		0.00001	g/L	estimated
Methanogen 2				
Cell mass	B_{m2}	1000	fg	(Sowers et al., 1984)
Mass of EPS capsule		10	fg	(Moletta et al., 1986)
Division radius		3	μm	(Sowers et al., 1984)
Maximum growth rate	$\widehat{\mu_{m2}}$	0.02	h^{-1}	(Gray et al., 2011)
Substrate saturation constant	K_{sH}	0.000018	g/L	(Gray et al., 2011)

Biomass conversion rate	α_{bh}	0.1	$\frac{g_{biomass}}{g_{hydrogen}}$	(Gray et al., 2011)
Substrate conversion rate	α_{mh}	2	$\frac{g_{methane}}{g_{hydrogen}}$	(Gray et al., 2011)
Death delay		144	h	estimated
Death threshold		0.000001	g/L	estimated

References

Angelidaki, I., Ellegaard, L., and Ahring, B. K. (1999). A comprehensive model of anaerobic bioconversion of complex substrates to biogas. *Biotechnol. Bioeng.* 63, 363–372.

Cussler, E. L. (2009). *Diffusion: mass transfer in fluid systems*. Cambridge University press.

Desvaux, M., Guedon, E., and Petitdemange, H. (2001). Carbon flux distribution and kinetics of cellulose fermentation in steady-state continuous cultures of *Clostridium cellulolyticum* on a chemically defined medium. *J. Bacteriol.* 183, 119–130. doi:10.1128/JB.183.1.119-130.2001.

Gavala, H. N., Angelidaki, I., and Ahring, B. K. (2003). “Kinetics and modeling of anaerobic digestion process,” in *Biomethanation I. Advances in Biochemical Engineering/Biotechnology*, ed. B. K. et al. Ahring (Berlin, Heidelberg: Springer), vol. 81. doi:https://doi.org/10.1007/3-540-45839-5_3.

Gray, N. D., Sherry, A., Grant, R. J., Rowan, A. K., Hubert, C. R. J., Callbeck, C. M., et al. (2011). The quantitative significance of *Syntrophaceae* and syntrophic partnerships in methanogenic degradation of crude oil alkanes. *Environ. Microbiol.* 13, 2957–2975. doi:10.1111/j.1462-2920.2011.02570.x.

Guedon, E., Payot, S., Desvaux, M., and Petitdemange, H. (1999). Carbon and electron flow in *Clostridium cellulolyticum* grown in chemostat culture on synthetic medium. *J. Bacteriol.* 181, 3262–3269. Available at: https://jb.asm.org/content/181/10/3262.

Haynes, W. (2012). *The CRC Handbook of Chemistry and Physics*. 93rd ed. Chemical Rubber Company.

Hazel, J. R., and Sidell, B. D. (1987). A method for the determination of diffusion coefficients for small molecules in aqueous solution. *Anal. Biochem.* 166, 335–341. doi:https://doi.org/10.1016/0003-2697(87)90582-3.

Hobbie, R. K., and Roth, B. J. (2007). *Intermediate physics for medicine and biology*. Springer Science & Business Media.

Ibba, M., and Fynn, G. H. (1991). Two stage methanogenesis of glucose byAcetogenium kivui and acetoclastic methanogenic Sp. *Biotechnol. Lett.* 13, 671–676. doi:10.1007/BF01086325.

Ihnat, M., and Goring, D. A. I. (1967). Shape of the cellodextrins in aqueous solution at 25 °C. *Can. J. Chem.* 45, 2353–2361. doi:10.1139/v67-382.

Kaksonen, A. H., Franzmann, P. D., and Puhakka, J. A. (2003). Performance and ethanol oxidation kinetics of a sulfate-reducing fluidized-bed reactor treating acidic metal-containing wastewater. *Biodegradation* 14, 207–217. doi:10.1023/A:1024262607099.

Kalyuzhnyi, S. V, and Davlyatshina, M. A. (1997). Batch anaerobic digestion of glucose and its mathematical modeling. I. Kinetic investigations. *Bioresour. Technol.* 59, 73–80. doi:https://doi.org/10.1016/S0960-8524(96)00124-1.

Koesnandar, Nishio, N., Kuroda, K., and Nagai, S. (1990). Methanogenesis of glucose by defined thermophilic coculture of *Clostridium thermoaceticum* and *Methanosarcina* sp. *J. Ferment. Bioeng.* 70, 398–403. doi:https://doi.org/10.1016/0922-338X(90)90121-C.

Kubitschek, H. E. (1990). Cell volume increase in *Escherichia coli* after shifts to richer media. *J. Bacteriol.* 172, 94–101. doi:10.1128/jb.172.1.94-101.1990.

Lens, P. N. L., Gastesi, R., Vergeldt, F., van Aelst, A. C., Pisabarro, A. G., and Van As, H. (2003). Diffusional properties of methanogenic granular sludge: 1H NMR characterization. *Appl. Environ. Microbiol.* 69, 6644–6649. doi:10.1128/AEM.69.11.6644-6649.2003.

Moletta, R., Verrier, D., and Albagnac, G. (1986). Dynamic modelling of anaerobic digestion. *Water Res.* 20, 427–434. doi:https://doi.org/10.1016/0043-1354(86)90189-2.

Nagpal, S., Chuichulcherm, S., Livingston, A., and Peeva, L. (2000). Ethanol utilization by sulfate-reducing bacteria: An experimental and modeling study. *Biotechnol. Bioeng.* 70, 533–543. doi:10.1002/1097-0290(20001205)70:5<533::AID-BIT8>3.0.CO;2-C.

Seitz, H.-J., Schink, B., Pfennig, N., and Conrad, R. (1990). Energetics of syntrophic ethanol oxidation in defined chemostat cocultures. *Arch. Microbiol.* 155, 82–88. doi:10.1007/BF00291279.

Sowers, K. R., Baron, S. F., and Ferry, J. G. (1984). *Methanosarcina acetivorans* sp. nov., an acetotrophic methane-producing bacterium isolated from marine sediments. *Appl. Environ. Microbiol.* 47, 971–978. Available at: https://pubmed.ncbi.nlm.nih.gov/16346552.

Stewart, J. M., Driedzic, W. R., and Berkelaar, J. A. (1991). Fatty-acid-binding protein facilitates the diffusion of oleate in a model cytosol system. *Biochem. J.* 275 (Pt 3), 569–573. doi:10.1042/bj2750569.

Tatton, M. J., Archer, D. B., Powell, G. E., and Parker, M. L. (1989). Methanogenesis from ethanol by defined mixed continuous cultures. *Appl. Environ. Microbiol.* 55, 440–445. Available at: https://pubmed.ncbi.nlm.nih.gov/16347852.

Yang, S.-T., and Tang, I.-C. (1991). Methanogenesis from lactate by a co-culture of *Clostridium formicoaceticum* and *Methanosarcina mazei*. *Appl. Microbiol. Biotechnol.* 35, 119–123. doi:10.1007/BF00180648.
